# Assessment of Personal Exposure to Particulate Air Pollution in Different Microenvironments and Traveling by Several Modes of Transportation in Bogotá, Colombia: Protocol for a Mixed Methods Study (ITHACA)

**DOI:** 10.2196/25690

**Published:** 2022-01-31

**Authors:** Jeadran N Malagon-Rojas, Diana Carolina Pinzón-Silva, Eliana L Parra, Luisa F Lagos M, Yesith Guillermo Toloza-Perez, Luis Jorge Hernández Florez, Ricardo Morales Betancourt, Sol Angie Romero, Ana Paola Ríos Cabra, Olga L Sarmiento

**Affiliations:** 1 Grupo de Salud Ambiental y Laboral Instituto Nacional de Salud Bogotá Colombia; 2 Facultad de Medicina Universidad El Bosque Bogotá Colombia; 3 Maestría en Epidemiologia Facultad de Medicina Universidad El Bosque Bogotá Colombia; 4 Grupo de Investigación Salud Pública Educación y Profesionalismo Universidad de los Andes Bogotá Colombia; 5 Centro de Investigación en Ingeniería Ambiental Universidad de los Andes Bogotá Colombia; 6 Grupo de Cuidado Cardiorrespiratorio Universidad Manuela Beltrán Bogotá Colombia; 7 Grupo de Epidemiología EPIANDES Universidad de los Andes Bogotá Colombia

**Keywords:** air pollution, particulate matter, black carbon, mixed methods, toxic, air quality, respiratory, pollution, pollutants, microenvironments, Bogota, respiratory disease, exposure to air pollutants, air contamination

## Abstract

**Background:**

Air pollution in most countries exceeds the levels recommended by the World Health Organization, causing up to one-third of deaths due to noncommunicable diseases. Fine particulate matter (PM_2.5_) and black carbon (BC) from mobile sources are the main contaminants.

**Objective:**

The aim of this study is to assess the relationship of exposure to air pollutants (PM_2.5_ and BC) in microenvironments according to respiratory health and physical activity in users traveling by different types of transportation in Bogotá, Colombia.

**Methods:**

A mixed methods study based on a convergent parallel design will be performed with workers and students. The sample will include 350 healthy transport users traveling by different urban transportation modes in three main routes in Bogotá. The study is broken down into two components: (1) a descriptive qualitative component focused on assessing the individual perception of air pollution using semistructured interviews; and (2) a cross-sectional study measuring the individual exposure to PM_2.5_ and BC using portable instruments (DustTrak and microAeth, respectively), pulmonary function by spirometry, and physical activity with accelerometry. The analysis will include concurrent triangulation and logistic regression.

**Results:**

The findings will be useful for the conception, design, and decision-making process in the sectors of health and mobility from public, academy, and private perspectives. This study includes personal measurements of PM_2.5_ and BC during typical trips in the city to assess the exposure to these contaminants in the major roadways in real time. The study further compares the performance of two different lung tests to identify possible short-term respiratory effects. As a limitation, the protocol will include participants from different institutions in the city, which are not necessarily representative of all healthy populations in Bogotá. In this sense, it is not possible to draw causation conclusions. Moreover, a convergent parallel design could be especially problematic concerning integration because such a design often lacks a clear plan for making a connection between the two sets of results, which may not be well connected. Nevertheless, this study adopts a procedure for how to integrate qualitative and quantitative data in the interpretation of the results and a multilevel regression. The time that participants must live in the city will be considered; this will be controlled in the stratified analysis. Another limitation is the wide age range and working status of the participants. Regional pollution levels and episodes (PM_2.5_) will be handled as confounding variables. The study is currently in the enrollment phase of the participants. Measurements have been made on 300 participants. Pandemic conditions affected the study schedule; however, the results are likely to be obtained by late 2022.

**Conclusions:**

This study investigates the exposure to air pollutants in microenvironments in Bogotá, Colombia. To our knowledge, this is the first mixed methods study focusing on PM_2.5_, BC, and respiratory health effects in a city over 2 meters above sea level. This study will provide an integration of air pollution exposure variables and respiratory health effects in different microenvironments.

**International Registered Report Identifier (IRRID):**

PRR1-10.2196/25690

## Introduction

Since the end of the 1950s, the world has experienced an exponential urban growth phenomenon [1]. The accelerated growth of cities has had a positive impact on the quality of life of citizens related to the supply of health, education, and work services, among other aspects. However, the growth of cities has also been accompanied by several emerging effects such as stress, mobility issues, and exposure to environmental pollutants [1,2]. To address these difficulties, a considerable number of cities worldwide have opted to promote citizens’ use of active transport such as walking and cycling, either alone or in combination with public transit [3]. This strategy, in principle, seems to be extraordinarily successful insofar as it promotes decongestion of the roads, which leads to improved air quality while promoting physical activity with benefits to cardiovascular health [4-7].

Studies have suggested that the health benefits of active transport are substantially higher than the negative effects associated with exposure to air pollution [8,9]. By contrast, other studies have demonstrated that the effects of exposure to particulate matter (PM) are considerable, particularly in the generation of cardiovascular and respiratory pathologies in the healthy population [10-20], which are especially evident in children under 5 years and adults over 65 years of age [21-24]. Numerous studies have provided information on the effects of air pollution on users of multiple modes of transport, including walking, bicycle, vehicle, and public transportation [24-27]. These studies grouped information from a considerable number of users of different modes of transport in cities, mainly in the United States and Europe [24-27]. In contrast, some studies carried out in Latin American cities have combined factors such as high rates of urbanization [28,29], the quality of the fuel used in the region [30,31], and active transport strategies [22,32-36]. Although such strategies are framed within an assumption of benefits related to physical activity, the effects of these approaches on exposure to air pollutants are not known.

In the last decade, some studies performed in the city of Bogotá, Colombia, have provided important information regarding the distribution of air pollutants in the city and the effects on respiratory health in children and occupational exposure to PM [37-41]. Nevertheless, these studies suffer from some limitations in not considering the sample size, the major roadways involved, and the measurement of air pollutants, among others [42,43]. It is important to highlight that the Secretaria Distrital de Movilidad of Bogotá has implemented a 10-year plan to improve mobility in the city, including a strategy to promote nonmotorized trips [44] and increase the number of bicycle users in the medium term. In this sense, this study will constitute a baseline to understand the relationship between exposure to air pollutants in different microenvironments and the potential short-term respiratory health effects on users.

The proposed study seeks to relate the exposure to fine particulate matter (<2.5 microns; PM_2.5_) and black carbon (BC) with the respiratory health and physical activity of healthy users who are mobilized in prioritized transportation microenvironments in Bogotá. The study has the following objectives: (1) to estimate the changes in lung volumes and respiratory symptoms of users according to the mode of transportation; (2) to determine the concentrations of PM_2.5_ and BC in the evaluated microenvironments; (3) to identify relationships among the variables of exposure to environmental pollutants, perception of air quality, and respiratory health in study participants; (4) to assess participants’ perceptions of air pollution in Bogotá; and (5) to measure physical activity in healthy volunteers.

## Methods

### Design

**Figure 1:**
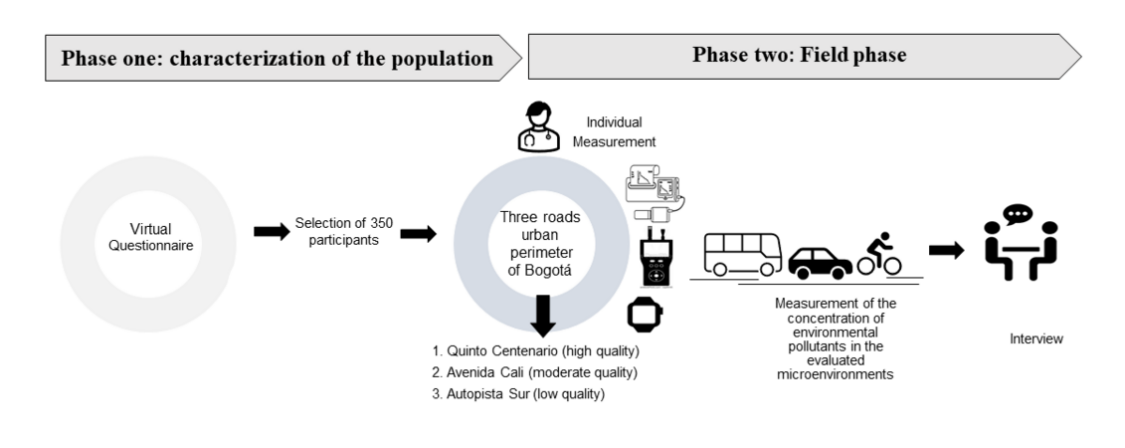
Diagram depicting the proposed process for measurement of the concentration of environmental pollutants in the evaluated microenvironments.

A mixed methods approach will be adopted using a convergent parallel design. The development of the study includes two phases: a phase of characterization of the population and a field phase (Figure 1). The objectives and methodologies to be used for the development of the study are summarized in Table 1.

**Table 1:** Objectives and methodologies to be used for the mixed methods study.

Objective		Methods	Techniques	Analysis
Phase 1: Sample selection		Quantitative	Electronic questionnaire	Descriptive
**Phase 2**				
	To describe the study participants’ perceptions of air pollution in Bogotá	Qualitative (descriptive)	Semistructured interview	Categorization and coding
	To measure physical activity in healthy volunteers	Quantitative (cross-sectional)	Measurement of vital signs, anthropometry, accelerometry (METs^a^)	Descriptive and bivariate
	To estimate the changes in lung volumes and respiratory symptoms of users according to the mode of transportation	Quantitative (cross-sectional)	Symptom questionnaire and spirometry	Descriptive and bivariate
	To determine the concentrations of PM_2.5_^b^ and BC^c^ in the evaluated microenvironments	Quantitative (cross-sectional)	PM_2.5_ and BC levels in real time	Descriptive and bivariate
	To identify relationships among the variables of exposure to environmental pollutants, perception of air quality, and respiratory health in study participants	Qualitative and quantitative	Triangulation	Triangulation

^a^MET: metabolic equivalent of task.

^b^PM_2.5_: fine particulate matter.

^c^BC: black carbon.

### Study Setting

Bogotá, the capital of Colombia, is located on the western slope of the eastern Cordillera of the Colombian Andes. The urban perimeter of Bogotá covers 37,945 hectares, which corresponds to 23.19% of the city. The average height of the urban perimeter is over 2600 meters above sea level. The average temperature is 14.4°C, with extreme values between –4°C and 25°C [45]. Bogotá has approximately 7,150,000 inhabitants, 52.2% of the population are women, with the majority aged between 16 and 50 years [46].

In 2006, Bogotá implemented a 10-year plan that focused on the promotion of active transport as part of the measures to improve mobility [44]. In this context, the use of nonmotorized transport, especially bicycles, has increased by 38%, from 611,000 trips in 2011 to 846,000 trips in 2015 [47].

### Sample Size and Sampling

A nonprobabilistic sampling approach will be carried out for consecutive cases (comparing two means). A one-sided hypothesis (Ha: A>B, Ho: A=B) will be tested as follows:

Ha: People exposed to higher levels of contamination have worse spirometry values.

Ho: People exposed to higher levels of contamination have the same spirometry values.

For an α error of 5% and β error of 10%, power (1–β) of 90%, and the one-sided hypothesis, the calculated K value is 8.6. The minimum significant magnitude of the difference (µ1–µ2) in forced vital capacity (FVC) was set to 0.28 [48,49]. The standard deviation (σ) in each group was calculated to be 1.2 [49]. Considering an estimated loss of 10%, the calculated sample size was 350 participants.

### Data Collection

This study will use questionnaires, semistructured interviews, medical evaluations, and personal air quality measurements to collect quantitative and qualitative data.

### Phase One: Characterization Questionnaire

An invitation will be sent to all potential volunteers at participating institutions. Government and educational entities will be included in the study. A virtual questionnaire (Multimedia Appendix 1) will be sent by mail to the participants. The questionnaire will ask for individual informed consent and will collect information on the sociodemographic and transportation usage behavior of the study population, which will further help the researchers to select potential participants for the second phase. The target sample size for this phase was estimated at 1200, which corresponds to 10% of the total population (12,000) belonging to the study entities.

### Phase Two: Field Measurements

A total of 350 participants will be selected from those participating in phase one of the study. The selection of the participants will be carried out by two researchers based on the inclusion/exclusion criteria. Subsequently, preselected participants will be contacted by phone. Participants will be picked up at their residence in vans that will take them to the initial point of the selected route. The mode of transport will be chosen by the participant.

### Inclusion and Exclusion Criteria

The study will include healthy men and women between 18 and 55 years old with a BMI <30; who were residents of the urban area of Bogotá in the last 12 months; and are students or workers that use a bicycle, public transport, Transmilenio, or a private vehicle from 7 to 10 AM to get around the city. Users of public transport or Transmilenio will be considered to use a mixed mode of transport since walking or another mode of transport is needed to get to the bus station. This information will be considered in the analysis.

**Figure 2:**
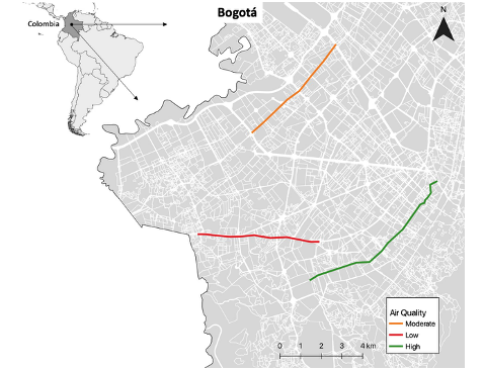
Three roads to be evaluated with a gradient of air quality from low to high.

Three roads will be evaluated, which are representative of a gradient in contamination: Quinto Centenario (high air quality), Avenida Cali (moderate air quality), and Autopista Sur (poor air quality) (Figure 2). These roads were chosen according to the level of historical contamination based on the records of the Air Quality Monitoring Network of Bogotá (Red de Monitoreo de Calidad del Aire de Bogotá [RMCAB]).

The study will exclude pregnant women; men and women with a chronic illness such as diabetes, asthma, chronic obstructive pulmonary disease, stroke, acute myocardial infarction, or deep vein thrombosis; and those with typical chest pain, fatigue, night sweats, or dyspnea on exertion in the last 6 months. Smokers or exsmokers who quit the habit within the past 12 months will also not be considered for participation.

### Participants’ Perception of Air Quality

From preestablished categories, semistructured guidelines will be formulated, including 10 questions, following the recommendations of DeJonckheere and Vaughn [50]. The guideline will be probed in a pilot with 5 volunteers, one for each transport mode. After traveling the route, participants will undergo a semistructured interview with an approximate duration of 30 minutes. All interviews will be conducted by trained study investigators. The interviews will be transcribed in Microsoft Word by one of the researchers. Interview transcripts and observation narratives will be coded thematically by two researchers independently. An online platform for qualitative content analysis (QCAmap) will be used [51] to generate codes according to preestablished categories [52] extracted from a literature review, including air quality perception [53,54], health affectation related to air pollution [55], and reasons for the use of transport modes [56].

### Spirometry-Based Estimation of Changes in Lung Volumes According to Mode of Transport

Spirometry tests will be performed and interpreted by a trained respiratory therapist following the recommendations of the Spanish Society of Pulmonology and Thoracic Surgery [57]. Spirometry will be performed before the start of the route and 2 hours after the end of the route [16]. The variables of the spirometry will be captured in a database. A survey (Multimedia Appendix 2) will also be used to evaluate the presence of respiratory symptoms after having completed the route [58].

### Estimation of Physical Activity Level

Physical activity levels will be estimated with a three-axis accelerometer (ActiGraph wGT3X-BT) using 60-second epochs and a sampling rate of 30-100 Hz. The accelerometer will be placed on the participant’s waist at the beginning of the travel route and will be removed at the end of the route. For participants traveling by bicycle, two accelerometers will be used with one placed on the waist and the other placed around the right ankle. Energy expenditure, measured as the metabolic equivalent of task (MET), will be estimated using ActiGraph software [59].

### Anthropometric Measurements

Measurements of weight and height will be taken for each participant according to the National Nutrition Situation Survey in Colombia [60]. To guarantee the precision of weight and height measurements, the same scale will be used throughout the study.

Before starting the trip, heart rate and baseline blood pressure will be measured using a digital tensiometer, according to the recommendations from the European Society of Hypertension Practice Guidelines for home blood pressure monitoring [61].

### Concentration of Environmental Pollutants in the Evaluated Microenvironments

Measurements of PM_2.5_ and BC will be taken along the participants’ routes. The duration of routes will consider the time reported in the Mobility Survey of Bogotá for 2019 [58]. To measure the PM_2.5_ levels, a portable photometer (Dustrak AM520) will be used throughout the travel [62]. The photometer will be in a backpack and the tube will be secured with a clip next to the participant’s neck. In this sense, the measurement of PM_2.5_ will most closely reflect the actual exposure level. To ensure the quality of the measurements obtained by portable photometers, calibration will be performed according to the recommendations from Betancourt et al [62].

To determine the concentration of BC, a portable aethalometer (MicroAeth AE51) will be used [63]. This measurement will be performed in real time and in the same manner as described for PM_2.5_ measurements. Before use, flow rate calibration of the aethalometer will be carried out following the manufacturer’s instructions and the recommendations of Betancourt et al [62].

### Determination of Potential Inhaled Dose

To estimate the potential inhaled dose of PM_2.5_, the following variables will be considered: the concentration of PM_2.5_ estimated for each mode of travel and roadway, the inhalation rate due to physical activity, and the exposure time to pollution, given in this case by the start and end time of each trip [62]. Three normalization factors will be used: dose per unit length, dose per unit time, and total dose [62].

### Data Analysis

#### Quantitative Analysis

A descriptive analysis will be carried out in R version 4.0.2 for Windows. In the characterization of the sample, the qualitative variables will be presented using absolute and relative frequencies. For continuous variables with a normal distribution, the mean and SD will be calculated; variables that do not present a normal distribution will be described with the median and 25th to 75th percentiles.

The Student *t* test with two tails and a repeated-measures analysis of variance will be applied according to the assumptions of each test for comparing the means of transport, with statistical significance determined at *P*<.05.

In addition to measuring exposure to air pollutants, the doses of inhaled air pollutants will be estimated to consider the differences in ventilation during cycling compared to traveling by car or bus. The inhaled dose is calculated by multiplying the concentrations of pollutants, ventilation per minute, and duration of the trip, divided by the body surface area. Linear mixed models will be performed on these data to analyze the effects of the concentrations and the inhaled doses of contamination of the air-related traffic on changes in lung function before and after exposure. The model will adjust for potential confounders, including age, gender, BMI, day of the week, time of measurement, location, mode of transportation, and travel time.

#### Qualitative Analysis

A content analysis will initially be carried out, segmented by participants’ characteristics (based on the mode of transport used). Next, categorization, and open, axial, and selective coding will be performed. From selective coding, attempts will be made to identify metaphors to identify the perceptions of air pollution [53-56].

#### Quantitative and Qualitative Results Integration

Since the study will be based on concurrent triangulation, in which the qualitative and quantitative data will be collected and analyzed simultaneously, we expect to have two sets of results that will be integrated into the overall interpretation for comparisons, which can help to improve understanding of the study problem.

Emergent categories from qualitative results will be compared with quantitative results to identify the extent to which they converge, diverge, or are related. Subsequently, the most frequent emergent categories will be transformed into a set of a categorical variable. These variables will be integrated into a multilevel model.

#### Multilevel Model

Owing to the hierarchical nature of the associations estimated in this study, an analysis of combined effects will be performed at various levels to adjust for the effect of exposure to air pollution on other variables associated with health. The first-level results of the individual variables will be provided: age, gender, travel mode, socioeconomic status, occupation, physical activity, respiratory symptoms score, BMI, heart rate, blood pressure, inhalation rate, FVC before and after travel, coefficient of variation for forced expiratory volume in the first second (FEV_1_) before and after travel, coefficient of variation for forced midexpiratory flow (FEF_25%-75%_), coefficient of variation for small airway reactance (R5), and concentrations of PM_2.5_ and BC [16]. The independent variables will be gender, age, occupation, place of study/work, socioeconomic status, air quality perception, average concentrations of PM_2.5_ and BC, inhaled dose, MET, and Tiffeneau index. A reduction in the FEF_25%-75%_ or an increase in the R5 coefficient of variation will be considered dependent variables. The microenvironment will be considered as a random variable and will be operationally defined by the combination of mode and route. Thus, there will be four possible modes and three routes, which provide 12 possible microenvironment combinations [64], resulting in the following level-1 model:

*Yij*=b0*j*+b1*j X1ij*+.......+b*nj Xnij*+*eij,*

where *Yij* is the dependent variable for participant *i* in unit *j*, b*nj* is the coefficient of level 1*, Xnij* is the explanatory variable *n* for participant *i* in unit *j, eij* is the random effect of level 1 (which is distributed normally with a mean of 0 and variance of σ^2^)*.*

The level-2 model is expressed as:

*bnj*=g*n*0+g*n*1 *W1j*+....+g*np Wpj*+*unj*,

where b*nj* is the dependent variable, g*n*0 and g*n*1 are the coefficients of level 1, g*np* is the coefficient of level 2*, Wpj* is the explanatory variable of level 2, and *unj* is the random effect of level 2; *u* is distributed in a normal multivalent manner with mean 0 and a matrix of variances.

### Ethical Considerations

The study was approved by the technical and ethical committee of the National Institute of Health (Instituto Nacional de Salud), as evidenced by approval protocol number 7, issued on April 4, 2019.

To ensure that participation will be voluntary, the invitation to participate in the study will be made through employers or faculties of the institutions to which individuals are enrolled. Consent will be obtained for each aspect of data collection as described above.

Electronic data, including transcribed questionnaire and interview responses, and data collected using portable devices and medical testing will be stored on password-protected and encrypted laptops.

Along with the questionnaire, medical evaluations and interviews will include collection of demographic and personal data; this information will contain no participant identifiers. The results from medical tests and air quality measures will be stored in a secure database. The data set will be analyzed only after the data collection phase. No identifying data on patients or participants will be collected.

### Patient and Public Involvement

This protocol was jointly developed between public and academic entities based on shared needs related to technical information for the promotion of policies that improve air quality in the city and reduce possible health effects. Some members of the Mesa Técnica Ciudadana por la Calidad del Aire de Bogotá were consulted as representatives of the citizenship, and their contributions were used to assign the participants to the road routes. Participants may voluntarily decide to participate in the study and may recommend participation in the study to others. Once data collection and analysis are complete, the results will be presented at academic events in addition to sharing with all participating entities.

## Results

Enrollment of participants has begun with a sample of 300 enrolled to date. The measures of inhaled doses have been taken along with air pollution measures. Data cleaning and management are ongoing. Initial results based on primary outcomes are expected to be disseminated by the end of 2022. The results could provide information on the perceptions of users with respect to the air quality to which they believe they are exposed considering various modes of transportation. Likewise, it is expected that the participants’ levels of physical activity and respiratory parameters may differ according to the different modes of transport, as well as with respect to variations in the amounts of PM_2.5_ and BC inhaled when traveling on routes with different levels of contamination, as established by environmental monitoring. The bivariate and logistic regression models are expected to establish relationships between the selected roads for the study and their characteristics at the levels of physical activity, pollution, and health effects for each microenvironment. These results should further provide a broader view of the problem of air quality in an active city such as Bogotá.

## Discussion

The objective of this study is to relate exposure to PM_2.5_ and BC with respiratory health and physical activity in a sample of healthy users mobilized in prioritized transportation microenvironments in Bogotá. The results of this study will allow us to establish a diagnosis of the effects of air pollution on the respiratory health of users of different modes of transport.

According to the report of the World Air Quality Index (IQAir AirVisual), among the 62 most polluted cities by PM_2.5_, Bogotá ranks in 44th position based on the 10 μg/m^3^ standard established by the World Health Organization [65]. Although the RMCAB reports data of contaminants as daily means, it is not possible to obtain a report of personal exposure in microenvironments [66,67]. Thus, the RMCAB network is inadequate for assessing personal exposure and cannot account for the measurement of BC [66].

This project will enable establishment of the pollution characteristics of PM_2.5_ and BC for some of the most important roads in the city, complementing the data produced by the RMCAB at the microenvironmental level. Likewise, this project will provide guidance to the health sector to focus actions for the prevention of respiratory diseases in the areas surrounding these roads, and will further guide the mobility sector in the layout of cycling routes with consideration of possible health impacts. The information provided by the study will inform the decision-makers: this study was formulated with collaborations of public policymakers on health, environment, and mobility in mind, in addition to considering the concerns of the Accidental Air Pollution Commission of the District Council. This ensures that the evidence generated is disseminated among public policymakers in the design of road infrastructure and active transport strategies to support evidence-based decision-making. Additionally, this study will allow participating work and study centers to learn about the health conditions and mobility of their population that could be used to implement plans for promoting physical activity in workers and students. Finally, this study will benefit the users of city transportation, who will be able to make autonomous decisions about the use of elements of personal protection, choice of routes, and means of transportation in the city using the information provided by the study.

This study has many limitations. The first limitation is related to the study design. Despite previous studies reporting changes in spirometry measures related to short-term exposure, such changes have not been reported in healthy populations living higher than 2000 meters above sea level. Second, to identify changes in spirometry parameters, the sample size is an important consideration. We have estimated a minimum sample size of 350 participants to guaranty the multilevel analysis requirements. Third, we will use the spirometric parameters to evaluate the health impacts of short-term exposure to air pollutants. However, the changes in the small airway may not be accurately measured by spirometry. We have considered alternative tests such as impulse oscillometry. Finally, in the qualitative analysis of the data, we will use a saturation criterion to define the number of semistructured interviews. This means that there will not be an interview for each participant, which can introduce bias in the multilevel model.

## Appendix

Multimedia Appendix 1 Virtual questionnaire.

Multimedia Appendix 2 Semistructured interview.

## Abbreviations

BC: black carbon
FEF25%-75%: forced midexpiratory flow
FEV1: forced expiratory volume in the first second
FVC: forced vital capacity
MET: metabolic equivalent of task
PM: particulate matter
PM_2.5_: fine particulate matter <2.5 microns
R5: small airway reactance
RMCAB: Red de Monitoreo de Calidad del Aire de Bogotá (Air Quality Monitoring Network of Bogotá)
