# Supplementary material for: Assessment of Personal Exposure to Particulate Air Pollution in Different Microenvironments and Traveling by Several Modes of Transportation in Bogotá, Colombia: Protocol for a Mixed Methods Study (ITHACA)
Source: JMIR Res Protoc. 2022 Jan 31;11(1):e25690. doi: 10.2196/25690 (PMC8845014; doi:10.2196/25690)
Supplement: Multimedia Appendix 1 [file resprot_v11i1e25690_app1.pdf]

Population characterization survey.

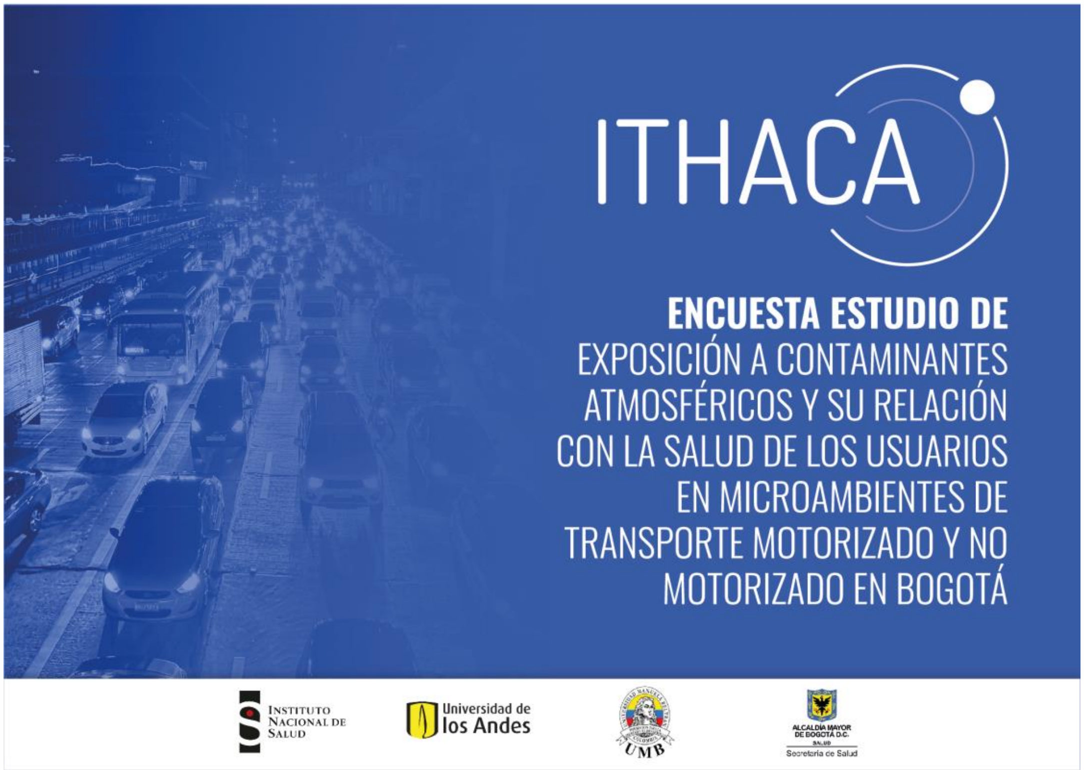

1. Email address

____

2. Phone number:

____

Informed consent to participate in the ITHACA project

This document provides you with the necessary information to participate voluntarily and freely in the research project carried out by the Instituto Nacional de Salud, in a group with the Universities of los Andes and Manuela Beltrán, and the District Health and Mobility Secretaries.

Before giving your consent, you need to fully understand the purpose of your decision. This process is called informed consent. Once you have read this section and voluntarily decide to participate in the study, you will be asked to accept at the end of this consent with a YES. Request that a copy of the survey is mailed to you.

Study justification: Air pollution is related to health alterations, especially the presence of ultrafine particles with a diameter less than 2.5 micrometers. In this way, this study asses in general overview of Bogotá air pollution and the health effects of being a user of motorized and non-motorized transport.

Purpose of the study: This study aims to relate exposure to atmospheric pollutants, its possible effect on health, and the perceptions that users have in micro-environments of motorized and non-motorized transport, about air pollution and health in the city of Bogotá

Method: Carry out an anonymized survey where you will be asked questions such as your age, socioeconomic status, sex, educational level, the mode of transportation you use, among others.

At any time, you can unilaterally withdraw from the study, without requiring a justification.

If you have questions or concerns, you can contact the principal investigator Ph.D Jeadran Malagón Rojas at the emails jmalagon@ins.gov.co or jnmalagon@unbosque.edu.co.

By accepting to participate in this survey you are giving your voluntary consent to participate in the characterization survey that is part of phase 1 of the ITHACA project: "Personal exposure to air pollutants and its relationship with health variables of the users in motorized and non-motorized transport microenvironments" and authorizes the researchers to:

3. Do you authorize the researchers to store the data provided in an anonymous database?

   Yes
   No

4. Do you authorize researchers to use the data provided for research purposes?

   Yes
   No

5. Do you authorize the researchers to contact you in the future for later phases of the study?

   Yes
   No

6. Do you want to actively participate in the project? Tours start at 5:30 am and end at 12:00 pm, therefore, you must have time available in that time slot. Participants are picked up at their place of residence, then we are transported to the starting point of the route to collect health data and begins with the tour accompanied by the researchers. On the way, personal exposure data will be collected. At the end of the tour, the second spirometry is taken, and the participants are again left at their place of residence.

   Yes
   No

7. Date of birth:

   ____

8. Have you lived in Bogotá for the last 12 months?

   Yes
   No

Socioeconomic Information

In this section you will find general questions about yourself, the place where you job or study, the transportation mode you use and the route you use to get around the city.

9. Gender:

   Female
   Male
   I prefer not to say
   Other

10. If your gender is female, are you pregnant?

   Yes
   No

11. Neighborhood where you live

____

12. Socioeconomic stratum that registers in the water bill

1
2
3
4
5
6
7

13. Main occupation (refers to the activity carried out mainly during the week). Currently, how is your work / study modality?

- Telecommuting Employee (includes telecommuting, job at home, etc.)
- On-site Employee (includes jobs for the provision of services, employment contract, part-time and others)
- Blended Employee
- Virtual student
- Blended student
- On-site student
- Unemployed
- Other

14. Institution in which you job or study related to the main occupation

- Instituto Nacional de Salud (INS)
- Universidad de los Andes
- Universidad Manuela Beltrán (UMB)
- Secretaría Distrital de Salud (SDS)
- Secretaría Distrital de Movilidad (SDM)
- Secretaría Distrital de Ambiente (SDA)
- Secretaría Distrital de Integración Social
- Instituto Distrital de Recreación y Deporte (IDRD)
- OPAIN S.A.
- Other

15. Educational level - Consider the last certified educational level you have

- Elementary School
- Incomplete Elementary School
- high school
- Incomplete high school
- Technique
- Incomplete technique

- Technological
- Incomplete technological
- Undergraduate
- Incomplete undergraduate
- Specialization
- Incomplete specialization
- master's degree
- Incomplete Mastery
- PhD
- Incomplete Ph.D.
- Postdoc

16. Locality where you live

- Usme
- San Cristóbal
- Rafael Uribe Uribe
- Bosa
- Tunjuelito
- Ciudad Bolívar
- Antonio Nariño
- Candelaria
- Santafe
- Mártires
- Kennedy
- Fontibón
- Puente Aranda
- Teusaquillo
- Chapinero
- Barrios Unidos
- Engativá
- Suba
- Usaquén
- Sumapaz
- Does not know

17. Location where you work or study (related to the main activity you previously listed).

- Usme
- San Cristóbal
- Rafael Uribe Uribe
- Bosa
- Tunjuelito
- Ciudad Bolívar
- Antonio Nariño
- Candelaria
- Santafe

- Mártires
- Kennedy
- Fontibón
- Puente Aranda
- Teusaquillo
- Chapinero
- Barrios Unidos
- Engativá
- Suba
- Usaquén
- Sumapaz
- Does not know
- Does not apply

18. If you leave your home, what is the approximate time in minutes it takes from your front door to the place of work / study?

____

19. If you leave your home, what is the time that you leave Monday through Friday to your place of work/study?

____

20. Since the Coronavirus pandemic, have you changed your work / study mode to?

- The mode does not change, I am still on-site
- Blended
- Virtual

21. The transport mode you used BEFORE THE COVID-19 PANDEMIC to move from your place of residence to the place of work/study (in which it lasted the longest) was:
    - Transmilenio
    - SITP - Urban (Blue)
    - Intermunicipal Bus
    - Taxi
    - Ladder bus/Chiva
    - Campero/Jeep
    - School transportation
    - Public bicycle
    - Private/company bus
    - Dual bus
    - Individual transport by van/car requested by mobile app

- SITP - Complementary (Orange)
- SITP - Special (purple)
- SITP - Provisional
- Informal car
- Taxi requested by app
- Collective taxi
- Bus/informal van/Chana
- Mototaxi
- Passenger cargo motorcar
- Animal-drawn vehicle
- Bicitaxi
- TransmiCable
- Train
- Bicycle
- Motor bike
- Motorcycle as a driver
- Motorcycle as passenger
- Private vehicle as driver
- Private vehicle as passenger
- Skateboards
- Human-powered vehicle
- Truck / Dump Truck / Tractomula
- Pedestrian
- BRT Feeder Bus
- Bicycle as a passenger

22. The transport mode you CURRENTLY use to get from your home to your Job/study site (which lasts the longest) is:
    - Transmilenio
    - SITP - Urban (Blue)
    - Intermunicipal Bus
    - Taxi
    - Ladder bus / Chiva
    - Campero / Jeep
    - School transportation
    - Public bicycle
    - Private/company bus
    - Dual bus
    - Individual transport by van/car requested by mobile app
    - SITP - Complementary (Orange)
    - SITP - Special (purple)
    - SITP - Provisional
    - Informal car
    - Taxi requested by app

- Collective taxi
- Bus /informal van/Chana
- Mototaxi
- Passenger cargo motorcar
- Animal-drawn vehicle
- Bicitaxi
- TransmiCable
- Train
- Bicycle
- Motor bike
- Motorcycle as a driver
- Motorcycle as passenger
- Private vehicle as driver
- Private vehicle as passenger
- Skateboards
- Human-powered vehicle
- Truck / Dump Truck / Tractomula
- Pedestrian
- BRT Feeder Bus
- Bicycle as a passenger

23. On the route you currently use to get to your place of job/study, do you use any of these avenues? - You can select more than one. Note: using refers to traveling along the avenue, not just crossing it
    - Ciudad de Cali Avenue
    - Ciudad de Quito Avenue (NQS-North Quito South)
    - El Dorado Avenue
    - Las Americas Avenue
    - Caracas Avenue
    - 17th Street (Chapinero)
    - 19th Street (Chapinero)
    - None of the options

24. Do you know how to ride a bicycle?
    Yes
    No

Medical history

Next you will be asked some questions about pathological history and habits

25. Have you smoked at least 100 cigarettes in your entire life?
    Yes
    No

26. How often did you smoke a cigarette in the last 30 days?

- Daily
- Some days
- Does not smoke

27. During the past 6 months (not including last month), did you smoke a cigarette daily?
    Yes
    No

28. How often did you smoke cigarettes during the last 6 months (not including the last month)?
    - Daily
    - Some days
    - Does not smoke

29. In the past 12 months, have you stopped smoking for a day or more because you were trying to quit?
    Yes
    No
    Does not apply

30. What is your weight in kilograms (kg) (without periods or commas)

    ____

31. What is your height in centimeters (cm) (without periods or commas)

    ____

32. Do you practice any sport, physical exercise, or moderate intensity activity that greatly increases your respiratory and heart rates (running or playing soccer) for at least 10 minutes at a time?
    Yes
    No

33. In a typical week, how many days do you do moderate-intensity activities, be it playing sports, exercising, or having fun?

    1 2 3 4 5 6 7

34. On a typical day, how much time do you spend doing moderate-intensity activities, be it playing sports, exercising, or having fun?

    ____

35. Before the COVID-19 pandemic, did you participate in any sport, physical exercise, or moderate intensity activity that increased your heart and breathing rate (running or playing soccer) for at least 10 minutes at a time?
    Yes
    No

36. Have you been diagnosed with any of the following illnesses? You can check more than one
    - COVID-19
    - Arterial hypertension
    - Chronic Obstructive Pulmonary Disease (COPD)
    - Renal disease
    - Cancer
    - Cerebrovascular event
    - Deep venous thrombosis
    - Asthma
    - Acute Myocardial Infarction
    - Mellitus diabetes
    - I do not have any sickness
    - Other: ___________

37. Do you take medicine for any of the following illnesses? You can check more than one:
    - COVID-19
    - Arterial hypertension
    - Chronic Obstructive Pulmonary Disease (COPD)
    - Renal disease
    - Cancer
    - Cerebrovascular event
    - Deep venous thrombosis
    - Asthma
    - Acute Myocardial Infarction
    - Mellitus diabetes
    - I do not have any sickness
    - Other: ___________

38. In case of having had COVID-19, what consequences did it leave?
    ____

39. How is your quality of life?

- Very bad
- Bad
- The normal

- Really good
- Very good

40. Overall, how satisfied are you with your health?
- Very unsatisfied
- Little satisfied
- The normal
- Quite satisfied
- Very satisfied

41. How satisfied are you with your job/study ability?
- Very unsatisfied
- Little satisfied
- The normal
- Quite satisfied
- Very satisfied

42. In general, the air quality you breathe on the way to the workplace/study is:
- Very bad
- Bad
- Regular
- Good
- Very good

43. How satisfied are you with your transportation mode?
- Very unsatisfied
- Little satisfied
- The normal
- Quite satisfied
- Very satisfied

44. How often do you have negative feelings (such as sadness, hopelessness, anxiety, depression)?
- Never
- Rarely
- Moderately
- Frequently
- Always

45. Are these negative feelings related to the situation generated by COVID-19?

    Yes

    No

46. Do you consider that there is a relationship between air quality and your state of health?
    Yes
    No

47. According to your perception, which are the organs or systems most affected by poor air quality in the city? You can check more than one if you want

- Respiratory system (Includes nose, larynx, pharynx, lungs)
- Skin and hair
- Cardiovascular system
- Gastrointestinal system
- Eyes
- None - does not affect at all
- Other: ____________

48. How do you know that the air you are breathing is polluted? You can check more than one option

- The air turns dark
- The air turns whitish
- The air has a characteristic odor

49. During the trips you make from home to job/ study, do you use anything or take any measures to protect yourself from air pollution?
    Yes
    No

50. If you have answered affirmatively to the previous question, mention which element you use to protect yourself:
    - Conventional mask
    - Mask N 95
    - Special filter
    - Close windows
    - Open windows
    - Hold the breath
    - I do not use any element
    - Other: __________

51. On a scale of 1 to 10, with 1 being the minimum exposure and 10 the maximum exposure, in your transport mode, how exposed do you feel to the Coronavirus?

    1 2 3 4 5 6 7 8 9 10
