# Supplementary material for: Assessment of Personal Exposure to Particulate Air Pollution in Different Microenvironments and Traveling by Several Modes of Transportation in Bogotá, Colombia: Protocol for a Mixed Methods Study (ITHACA)
Source: JMIR Res Protoc. 2022 Jan 31;11(1):e25690. doi: 10.2196/25690 (PMC8845014; doi:10.2196/25690)
Supplement: Multimedia Appendix 2 [file resprot_v11i1e25690_app2.pdf]

Semi-structured interview

The aim of this semi-structured interview is to collect information about knowledge, attitudes and practices related to the perception of air quality of people who live and carry out their activities in Bogotá.

The interview will be recorded in Digital Voice Recorder and will last approximately between 20 and 30 minutes.

The interview will be carried out individually with people who travel through each of the three routes as regular users of a bicycle, private vehicle, or public transport.

The transcription of the interviews will be textual and will be made after the interview; the entire audio will be transcribed.

The questions to be asked are presented below:

Number of participants______ Gender______ Date_______

Mode of transport__________ Route_______ Age_______

Knowledge

¿What perception do you have of the air quality in Bogotá?

¿What do you consider to be the main problem related to pollution?

¿Do you know the risks faced by people who travel in polluted environments?

¿What are the effects on the health of people who travel in contaminated environments and how are they evidenced?

Attitudes:

¿To what extent does air quality interfere with your quality of life?

¿What attitude do you take when you are exposed to a direct source of contamination?

¿Do you think you have become ill because of the exposure to environmental pollutants? ¿Which was the disease? ¿Did your attitude change from this episode?

Practices:

¿Do you consider that you take care of yourself from exposure to environmental pollutants? How?

¿Do you consider that you protect yourself against exposure to environmental pollutants? How?

¿What kind of activities do you perform to take care of your health?

¿How do you think the city's air quality could be improved?
